# Targeted assemblies of *cas1* suggest CRISPR-Cas’s response to soil warming

**DOI:** 10.1038/s41396-020-0635-1

**Published:** 2020-03-27

**Authors:** Ruonan Wu, Benli Chai, James R. Cole, Santosh K. Gunturu, Xue Guo, Renmao Tian, Ji-Dong Gu, Jizhong Zhou, James M. Tiedje

**Affiliations:** 1 Laboratory of Environmental Microbiology and Toxicology, School of Biological Sciences, Faculty of Science, The University of Hong Kong, Hong Kong SAR, China; 2 Center for Microbial Ecology, Michigan State University, East Lansing, MI USA; 3 Department of Plant, Soil and Microbial Sciences, Michigan State University, East Lansing, MI USA; 4 Department of Microbiology & Plant Biology, Institute for Environmental Genomics, and School of Civil Engineering and Environmental Sciences, University of Oklahoma, Norman, OK USA; 5 State Key Joint Laboratory of Environment Simulation and Pollution Control, School of Environment, Tsinghua University, Beijing, China; 6 Institute for Food Safety and Health, Illinois Institute of Technology, Chicago, IL USA; 7 Earth and Environmental Sciences, Lawrence Berkeley National Laboratory, Berkeley, CA USA

**Keywords:** Microbial ecology, Climate-change ecology

## Abstract

There is an increasing interest in the clustered regularly interspaced short palindromic repeats CRISPR-associated protein (CRISPR-Cas) system to reveal potential virus–host dynamics. The universal and most conserved Cas protein, *cas1* is an ideal marker to elucidate CRISPR-Cas ecology. We constructed eight Hidden Markov Models (HMMs) and assembled *cas1* directly from metagenomes by a targeted-gene assembler, Xander, to improve detection capacity and resolve the diverse CRISPR-Cas systems. The eight HMMs were first validated by recovering all 17 *cas1* subtypes from the simulated metagenome generated from 91 prokaryotic genomes across 11 phyla. We challenged the targeted method with 48 metagenomes from a tallgrass prairie in Central Oklahoma recovering 3394 *cas1*. Among those, 88 were near full length, 5 times more than in de-novo assemblies from the Oklahoma metagenomes. To validate the host assignment by *cas1*, the targeted-assembled *cas1* was mapped to the de-novo assembled contigs. All the phylum assignments of those mapped contigs were assigned independent of CRISPR-Cas genes on the same contigs and consistent with the host taxonomies predicted by the mapped *cas1*. We then investigated whether 8 years of soil warming altered *cas1* prevalence within the communities. A shift in microbial abundances was observed during the year with the biggest temperature differential (mean 4.16 °C above ambient). *cas1* prevalence increased and even in the phyla with decreased microbial abundances over the next 3 years, suggesting increasing virus–host interactions in response to soil warming. This targeted method provides an alternative means to effectively mine *cas1* from metagenomes and uncover the host communities.

## Introduction

CRISPR-Cas system is mainly known as an adaptive immunity that enables the bacterial and archaeal hosts to robustly adapt to the rapidly evolving viruses by acquiring viral sequences and storing in CRISPR arrays as immunity memories [1]. Our knowledge of CRISPR-Cas system is restricted to a limited number of archaeal and bacterial genomes deposited in the public databases which cannot well-represent environmental microbiomes. A few pilot metagenome studies detected new CRISPR-Cas systems and their prevalence in the environments like acid mine drainage [2], sediments [2], marine sponge [3], and global oceans [4]. The CRISPR-Cas system in environmental microbiomes, such in soil, with high microbial diversity, and cryptic species are understudied. Some basic questions remain unanswered such as: (i) what are the subtypes and the host community of CRISPR-Cas system found in soil microbiome, and (ii) how does the CRISPR-Cas preference shift in response to environmental perturbation.

Compared with CRISPR arrays, *cas* genes provide more information of subtypes and host taxonomy [5]. Current methods of mining for *cas* genes in metagenome are based on the de-novo assembled contigs and binned genomes [6]. Assembling long contigs requires high coverage of the genomes so that the dominant microbes with low genomic heterogeneity and less repetitive regions are often more represented in de-novo assemblies [7]. Besides, the incomplete assembling of soil metagenomes generates shorter contigs, which makes *cas* gene annotation more problematic. In addition to the computational challenges, the viral load in soil is much lower than that in marine system, 1.5 × 10^8^ g^−1^, which is approximately one soil virus for every 25 bacterial cells [8] in contrast to 0.03–11.71 × 10^9^ g^−1^ viruses found in marine sediments [9]. Given the potentially low recovery of CRISPR-Cas system in soil metagenomes by current approaches, alternative methods are required.

To overcome the difficulties mentioned above, we applied a more targeted approach to assemble *cas* directly from the metagenomes by a targeted-gene assembler, Xander [10] using profile Hidden Markov Models (HMMs) to guide de Bruijn graph traversal. This could provide an opportunity to more efficiently reveal CRISPR-Cas systems in complex microbiomes such as in soil. We targeted *cas1*, one of the universally conserved *cas* genes, to assemble as a biomarker of CRISPR-Cas system [1]. Cas1 binds to Cas2 to form a complex to initiate the adaptive immunity at the integration stage in the majority of CRISPR-Cas systems [11]. An exception is putative CRISPR-Cas subtype IV, lacking *cas1*, *cas2*, and CRISPR arrays, is carried by a plasmid and opportunistically becomes functional only when transferred into hosts with CRISPR available, which is not included in the detection here. Hence, we applied this targeted-assembly method to more robustly recover *cas1* diversity and decipher CRISPR-Cas ecology.

Different soil histories or exposures to new environmental conditions drive soil microbiome changes, which could include their relationships with their viral predators. Climate change which can result in warmer soils, as well as new moisture regimes is one example of an environmental driver of microbiome change of current concern. In situ experimental soil warming studies have been conducted in various ecosystems including temperate forest soils [12, 13], temperate grassland soils [14, 15], high latitude alpine meadows [16], Antarctic peninsula [17], and Alaska tundra [18, 19], among which microbial succession and their ecological functions have been investigated but not potential interactions between prokaryotes and viruses. *cas1* may reflect the dynamics of this interaction in response to soil warming. This may show whether or how soil warming affects microbial communities and also shed light on the microbial response to environmental changes.

## Materials and methods

### Site description and soil property measurements

The soil warming experiment was set up at the Kessler Atmospheric and Ecological Field Station in McClain County, OK (34° 59′ N, 96° 31′ W). The mean annual temperature is 16.3 °C and mean annual precipitation is 914 mm [20]. The experiment was a blocked split-plot design and initiated in July, 2009. For each selected block, soil was sampled yearly through 2016 from three randomly selected warming replicates heated by above ground infrared radiators and from three paired ambient control plots located 5 m away. The sampling scheme, soil properties measurements, and DNA extraction from 48 soil samples used (2 treatments, 3 replicates over 8 years) were reported previously [15, 21].

### Constructing Cas1 HMMs and Xander packages

The three files for automatically generating a Xander package include (1) a fasta file containing seed sequences; (2) an HMM, and (3) a fasta file containing a more diverse collection of the targeted protein sequences (framebot.fa). Near full length of *cas1* protein sequences were retrieved from GenBank [22] for the well-curated protein family seed sequences in Pfam [23] (Pfam 01867), TIGERFAM [24] (TIGRFAM00287, TIGRFAM03637, TIGRFAM03638, TIGRFAM03639, TIGRFAM03640, TIGRFAM03641, TIGRFAM03983, TIGRFAM04093, and TIGRFAM04329) and the recent literature [2, 25–29]. The compiled *cas1* protein sequences were aligned by MAFFT [30] in Jalview (jalview.org, v. 2.10.2b2). The aligned *cas1* protein sequences were dereplicated to remove the identical or substring of sequences and clustered by sequence similarity using RDPTools (ReadSeq.jar and Clustering.jar; https://github.com/rdpstaff/RDPTools). The dereplicated *cas1* protein sequences grouped into seven complete-linkage clusters at 50% identity cutoff. The sequences in each cluster were used as the seed sequences to build HMMs using modified HMMER 3.0 [31] with a patch file [10]. An additional HMM was added specifically for archaeal Type II *cas1* considering its novelty [2]. To prepare the third file required for a Xander package (framebot.fa), we used the respective HMM to search against the nonredundant protein sequence database (nr, NCBI) via *hmmsearch* [31] and collected sequences annotated as ‘Cas1’. Eight *cas1* Xander packages named as M1–M8 were automatically prepared using a shell script (https://github.com/rdpstaff/Xander_assembler/blob/master/bin/).

### Evaluating and improving HMM performance

**Fig. 1:**
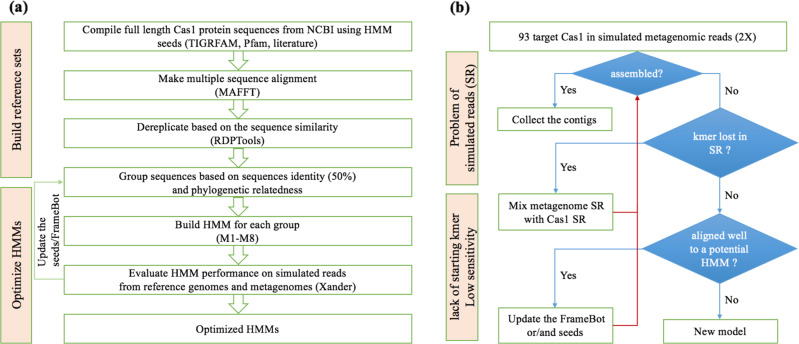
The procedures of HMM construction and model optimization. HMM construction (**a**): near full length of *cas1* protein sequences previously used in Cas1 TIGRFAM, Pfam and mentioned in the literature were retrieved from NCBI and were cluster at 50% sequence identity after aligned by MAFFT and dereplicated by RDPTools. Eight HMMs were constructed based on the seed sequences in each cluster. Model optimization (**b**): the HMM performance was evaluated by the simulated reads generated from a set of reference genomes carrying 17 subtypes of CRISPR-Cas systems. We optimized the HMMs by updating the coverage of the corresponding seed sequences and Framebot files.

We created a reference genome set including 91 bacterial and archaeal genomes across 11 phyla carrying 93 *cas1* sequences of 17 subtypes from NCBI Genome database (https://www.ncbi.nlm.nih.gov/genome) (Supplementary T1) to evaluate and train the newly constructed HMMs (Fig. 1b). The subtypes included Archaeal Type II, CasX, CasY, IA-IF, IU, IIA-IIC, and IIIA-IIID, which are classified followed the naming standard that has been specified by Makarova KS and Koonin EV [5]. Pairwise distances among the 93 *cas1* protein sequences were calculated (https://github.com/rdpstaff/Clustering), and a *cas1* protein tree was built by FastTree [32]. Together with the reference genome set, a genome of *Streptomyces coelicolor* (NC_003888.3) with a gene encoding transposase, a homolog of *cas1* protein [33], was included as the outgroup to root the *cas1* protein tree and also used to detect potential false positives in the following evaluation step.

First, we used the reference genome set to evaluate the sensitivity and specificity of the eight HMMs. Simulated metagenomic reads from the 91 genomes were generated by an amplicon and shotgun sequence simulator, Grinder [34], starting with a 2× coverage of each genome. The simulated metagenome was then fed to Xander with the eight Xander packages to assemble *cas1*. The assemblies were then subjected to chimera check by Xander. The HMM sensitivity was assessed by whether all the 17 subtypes of *cas1* can be recovered from the simulated metagenome using the targeted-assembly method. The HMM specificity was evaluated by accurate mapping of the targeted-assembled nucleotide sequences to the *cas1* coding region of the designated genome from the reference genome set using Bowtie2 [35]. The respective *cas1* protein sequences were also searched against nr database (NCBI) by BLAST [36] to check the assembly accuracy.

Second, the curated reference genome set can better improve the performance of the eight HMMs within the Xander packages. We first confirmed that the *cas1* sequences of the reference genomes were covered in the simulated metagenome by checking if at least two 45-mer of the reference *cas1* was in the kmer set of simulated reads (Fig. 1b). For the reference *cas1* that contributed to the simulated reads but was not captured by any of the preliminary models, we aligned the corresponding protein sequence of the reference *cas1* to the eight HMMs and added it to the seeds and framebot.fa of the best aligned HMM to enhance the sensitivity and the recovery of *cas1* diversity (Fig. 1b).

Similar to the simulated metagenomes, the preliminary assemblies of *cas1* proteins from the Oklahoma soil metagenomes mentioned below were also used to improve the performance of HMMs for targeted assembly. We searched the preliminary *cas1* assemblies (though short and below the length threshold) against nr database (NCBI) by BLAST [36] and added the near full-length best hits into the corresponding seeds and framebot files to optimize the models.

After the iteration by updating the coverage of the seeds and framebot files (Fig. 1a) and reconstructing the HMMs, the eight final Xander packages contain more robust models, which are available at https://github.com/Ruonan0101/Targeted_Cas1_assembly. The processing steps are summarized in Fig. 1a.

### Sequence trimming and targeted *cas1* assembly from Oklahoma soil metagenomes

The Illumina adapters were removed from the paired-end reads using Trimmomatic v0.33 [37] (ILUMINACLIP). The trimmed reads were filtered for contiguous segments longer than 45 bases (same as the kmer length used in assembling) with the average quality score higher than 20 (SolexaQA++ v3.1.5 [38] dynamictrim and lengthsort). The trimmed sequences were fed to Xander [10] for targeted *cas1* assembly. All eight *cas1* HMMs, together with bacterial and archaeal ribosomal protein L2 (*rplB*) HMMs, were run simultaneously on each sample from the same bloom filter with the same kmer size of 45 since this larger kmer size can result in better assemblies with lower chimera occurrence. The length cutoff for *cas1* protein sequences was set to 60 amino acids based on the lowest length coverage of *cas1* subtype, which was IIID at 30.8% (Table 1). We kept *rplB* sequences longer than 150 amino acids. RplB was used rather than the 16S rRNA gene because it is a single copy ribosomal protein in all microbes and is well tested for assembly by Xander and taxonomic placement [39].

**Table 1:** *cas1* subtypes included in the training set, and subtype coverage and length recovered by each model.

Subtype covered			No. of Cas1 included				Model		Average coverage (length)			Coverage (subtype)					
IF			8				M1		92.3%			100.0%					
IE			20				M2		86.2%			100.0%					
CasY			3				M3		99.6%			100.0%					
IIC/IIA/IIIA			3/5/2				M4		94.9%			100.0%					
IA/IIB/IIIB/CasX			5/2/2/1				M5		59.0%			100.0%					
Archaeal II			1				M6		99.4%			100.0%					
IC/ID/IU/IIIC/IIID			17/2/3/3/2				M7		82.7%			100.0%					
IB			14				M8		86.9%			100.0%					

Cas1 subtypes	Archaeal II	CasX	CasY	IA	IB	IC	ID	IE	IF	IU	IIA	IIB	IIC	IIIA	IIIB	IIIC	IIID
Average coverage (length)	99.4%	40.4%	99.6%	31.5%	85.5%	97.1%	98.2%	86.2%	92.3%	60.4%	95.7%	91.1%	95.4%	96.4%	99.4%	72.7%	30.8%
Coverage (subtype)	100.0%	100.0%	100.0%	100.0%	100.0%	100.0%	100.0%	100.0%	100.0%	100.0%	100.0%	100.0%	100.0%	100.0%	100.0%	100.0%	100.0%

### Contigs validation and host assignment to phylum level

Because the eight HMMs have overlapping specificity, we first dereplicated the combined assemblies from all eight models before predicting host taxonomy. All contigs from one sample were pooled together and searched against NCBI nr database. The top five hits with the descending bit scores of each contig were recorded together with the information of percent identity, coverage, accession number, and lineage. The best hit was selected only if the percent identity of the top hit was greater than the second one by 3% or it has the same host taxonomy with the remaining hits (phyla level used in the following data analysis), otherwise the contig was unclassified. The unclassified contigs kept the information of the best hit but with lineage noted as “unclassified.” All the classified and unclassified contigs were sorted based on accession numbers of the best hit to check the potential overlap between models. Contigs assembled by different models but with the same accession number of the best hit were re-examined and the ones with (1) classified lineage, (2) higher bit score, and (3) longer length were selected. The host of *cas1* assemblies was assigned based on the taxonomy annotation of the screened hits.

### Validation of assembly accuracy by comparing to the traditional annotation method

We leveraged de-novo assembled contigs obtained from Oklahoma grassland metagenomes to validate the assembly accuracy and evaluate the performance of the targeted method. The quality-filtered metagenomic reads were assembled by MegaHit [40] using kmer from 31 to 131 with step size of 20. The option of -kmin-1pass was used to recover low coverage species. There were 1,488,684 contigs with length longer than 1000 bp used for the following analysis.

We then applied the traditional *cas1* annotation method by searching the ORFs predicted from all the de-novo assembled contigs using the eight HMMs. The ORFs annotated as putative *cas1* via *hmmsearch* (HMMER, v3.1b2, http://hmmer.org/) were further validated by NCBI nr database. As both targeted and de-novo assembly methods can generate gene fragments, we only compared the numbers of *cas1* sequences with near full length (>300 aa) from both methods to make a fair and conservative estimate of the performance.

### Validation of host assignment method by comparing to the *cas1*-mapped de-novo assemblies

The reliability of *cas1*-based host assignment was tested by searching the targeted-assembled *cas1* sequences against the de-novo assembled contigs using BLASTn. A qualified match was defined as a hit with (1) the highest bit score, (2) the percent identity greater than 95%, and (3) the length coverage higher than 50%. We predicted the taxonomies of de-novo assembled contigs via the Contig Annotation Tool (CAT) [41] with modifications. CAT assigned a de-novo assembled contigs with NCBI lineage based on multiple open reading frames (ORFs) predicted by Prodigal [42]. As *cas1* is normally grouped with other CRISPR-Cas genes, which are more prone to horizontal transfer [1], we removed ORFs with NCBI annotations as CRISPR-Cas related proteins and fed CAT with filtered ORFs for taxonomy assignment. To check the sequence redundancy of *cas1*-mapped de-novo assembled contigs, we used the single linkage clustering to cluster the de-novo assembled contigs [43]. The detailed clustering method and results were in Supplementary File 1.

### *cas1* subtype assignment and prevalence estimation

As *cas1* protein sequences were mostly grouped by subtype, we assigned the subtypes by placing the assemblies on the *cas1* protein tree. The tree was built with the 93 *cas1* protein sequences of 17 subtypes from the reference genome set using FastTree [32]. The *cas1* assemblies filtered from the validation step were aligned to a reference HMM and the modeled positions were used to map to the branches of the *cas1* protein tree via pplacer [44]. The *cas1* contigs then adopt the subtype assignment of the mapped references.

*cas1* abundance was adjusted by subtracting the abundance of the contigs assembled from the potentially overlapped models. The adjusted *cas1* abundance detected in a microbial phylum was further normalized by the corresponding *rplB* abundance and noted as *cas1* prevalence of a particular phylum in the following discussion.

### Statistical analysis

Sequence distance matrix was calculated using RDPTools (Clustering) and plotted in R (v3.3.3, Vegan, pheatmap [45]). Canonical correspondence analysis including the *cas1* host composition with environmental attributes was conducted by R (v3.3.3) with packages of Vegan [46] and ggplot2 [47].

## Results

### Targeted-assembly method provides a high coverage of cas1 diversity

**Fig. 2:**
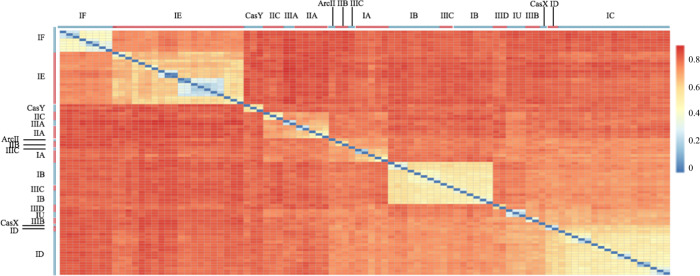
Pairwise distances among *cas1* protein sequences in the reference set of 91 genomes. The designation of the 17 subtypes is shown. The scale of dissimilarity is from 0 to 1. The hotter color indicates a higher dissimilarity.

To assess *cas1* diversity, the pairwise distance of the 93 *cas1* protein sequences belonging to 17 subtypes in the curated reference genome set (Fig. 2) was analyzed. The same subtype showed higher similarities but the distances within subtype can be up to 50–60%. The distance between different subtypes can be up to 80%. These revealed a high *cas1* protein sequence diversity. Although HMMs are known to robustly detect remote protein homology [48], multiple HMMs were need to cover *cas1* diversity. A limited (but sufficient) number of HMMs is preferred to avoid the potential coverage overlaps among models.

To test the coverages of the eight optimized HMMs implemented in Xander, we first assembled all the 17 subtypes of *cas1* from the simulated metagenomes (Table 1). Generally, the eight HMMs have high average length coverages of targeted *cas1* protein sequences (59–99% length coverage). Owing to the high diversity of some *cas1* sequences, subtypes like CasX, IA, and IIID could be recovered by 30–40% of their length.

**Fig. 3:**
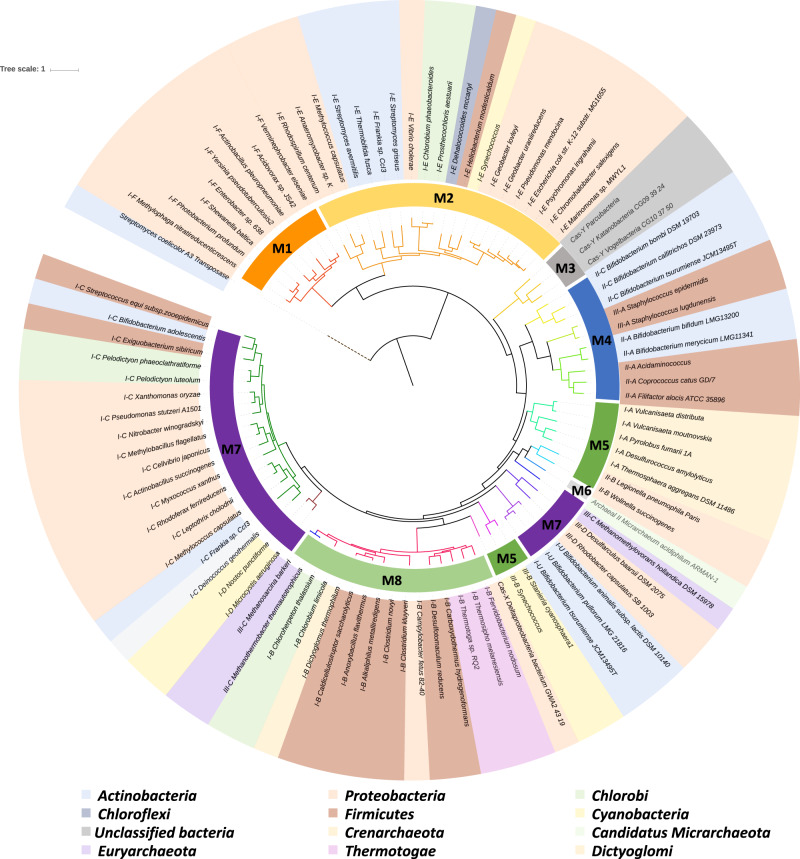
Rooted phylogenetic tree of *cas1* protein sequences from the reference genomes. Clades formed by 17 subtypes are colored differently. Model (M1–M8) coverages on different subtypes are shown in the inner circle and the taxonomy (phylum) of the host is displayed in the outer circle.

We built a *cas1* protein tree (Fig. 3) to visualize the clustering of the different subtypes and potential coverage overlap among the eight models. *cas1* protein sequences were clustered by subtypes and within each subtype, those from the same phylum generally clustered together (Fig. 3). Most of the models covered one clade except M5 and M7 (Fig. 3). *cas1* protein sequences covered by M5 and M7 were both developed a deep subclade, which intersected with each other, implying potential overlap between models (Fig. 3). The Bowtie results (Supplementary T2) of the assemblies from the simulated metagenome generated from the reference genomes indeed showed some M7-assembled contigs were also captured by M5 but not the opposite. In this case, M7 always gave longer contigs. Length sorting was therefore incorporated in the contig validation step to remove the replicated assemblies resulting from the models with overlapping coverage.

### Targeted method increased the detection capacity and reliably resolved *cas1* host community

To evaluate the performance of the targeted method and validate host assignment using the targeted-assembled *cas1*, we cross-checked *cas1* annotated from contigs that were de-novo assembled from the Oklahoma soil metagenomes analyzed below.

Compared with 88 dereplicated near full length (greater than 300 amino acids) targeted *cas1* assemblies, there were 17 near full length dereplicated *cas1* genes from the de-novo assembled contigs (Supplementary T3). Nine of these matched targeted *cas1* assemblies at greater than 99% nucleotide sequence identity. The other eight were distant to the known *cas1* sequences, with between 45% and 83% amino acid identity to their closest *cas1* BLAST matches. In comparison with the traditional method, the targeted method increased the detection capacity of recovering near full-length *cas1* by five times (88 in targeted method versus 17 in traditional method). In addition, ~3300 targeted-assembled *cas1* shorter than 300 amino acids were not included in this comparison but also contribute to *cas1* diversity that can only be recovered by the targeted method.

The de-novo assemblies were also used to test the reliability of *cas1* host assignment. We used BLAST to find contigs matching the targeted *cas1* assemblies and found 147 de-novo assembled contigs with BLAST matches to 27 different targeted-assembled *cas1*. All but three of these have a match with greater than 99% nucleotide identity (Supplementary T4). These de-novo contigs could be dereplicated into 25 clusters with 100% amino acid pairwise identity between the shared ORFs, but for host assignment each contig was assessed individually. Among the 147 *cas1*-mapped de-novo assembled contigs, 110 (74%) could be assigned to host phyla using the CAT program after removing putative CRISPR-Cas genes. All the assignments agreed with the matching targeted-assembled *cas1* assignment (Supplementary T4).

### *cas1* host community and subtypes in Oklahoma grassland microbiomes

**Fig. 4:**
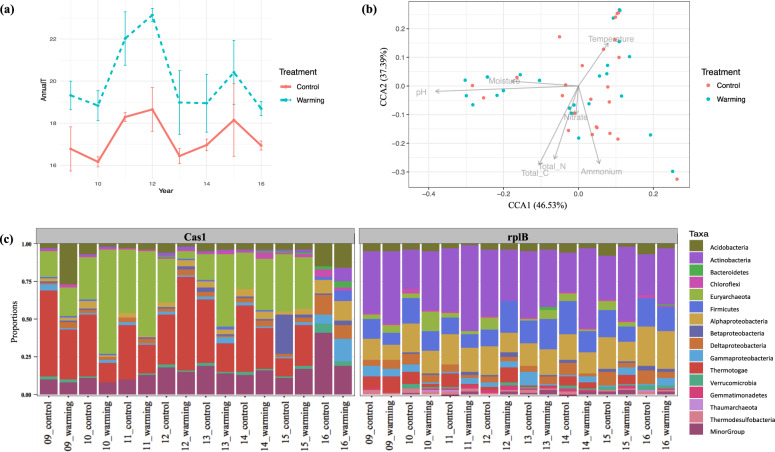
Increasing temperature with 8-year warming treatment and *cas1* host composition. Temperature fluctuation in warming and paired control treatment (**a**); canonical correspondence analyses (CCA) of *cas1* host composition with the environmental attributes (**b**); *cas1* host composition (**c**, left panel) and microbial composition revealed by *rplB* (**c**, right panel).

**Fig. 5:**
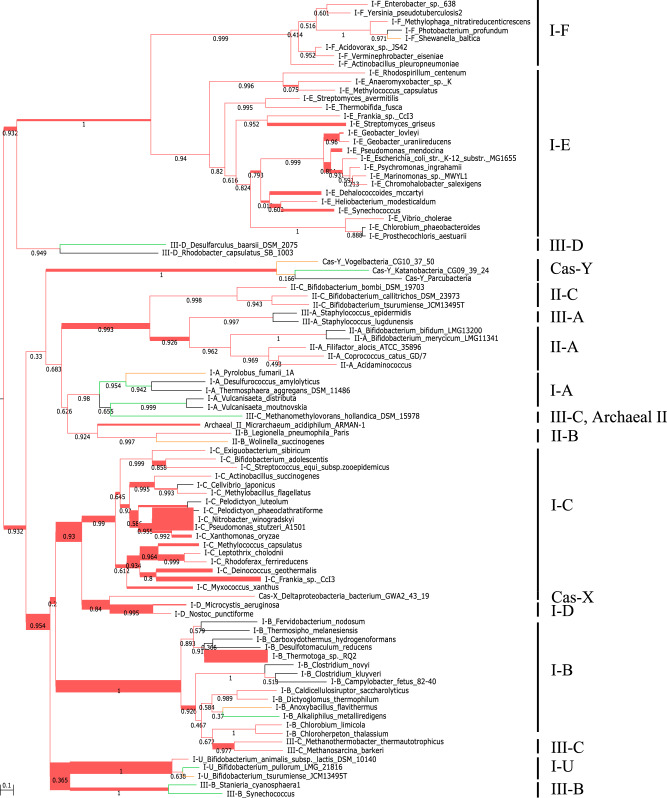
Subtype annotation of *cas1* protein assemblies from control and warming treatments. *cas1* protein sequences assembled from Oklahoma soil metagenomes with control and warming treatments were mapped to the branches of Cas1 reference tree that was constructed by 93 *cas1* protein sequences with the verified subtypes specified on the right. The branches placed with *cas1* protein sequences assembled from both control and warming samples are highlighted in red. The branches mapped by *cas1* protein sequences from control or warming samples are colored in green and yellow, respectively.

The 8-year continuous soil warming significantly increased the annual soil temperature above ambient by a mean of 2.38 °C with the largest increase of 4.16 °C, which occurred in the 4th year, 2012 (Fig. 4a). We targeted-assembled *rplB* and *cas1* from 48 Oklahoma soil metagenomes with the average reads count of 1.0 × 10^8^. The single copy core gene, *rplB* was used as a phylum-specific marker to estimate the microbial abundances and the major soil taxa were noted as the ones with *rplB* relative abundances greater than 1% (Fig. 4c, *rplB*). About 60% of the total *cas1* counts were detected in the major phyla, suggesting *cas1* was mainly distributed in the abundant phyla (Fig. 4c, Cas1). As noted above, *cas1* was prevalent in *Euryarchaota* and *Thermotogae* (Fig. 4c, Cas1). *cas1* host composition slightly changed in response to soil warming (Fig. 4c, Cas1). Besides temperature, *cas1* host composition was under the impacts of nutrients (total carbon, total nitrogen, ammonium, and nitrate), pH and moisture (Fig. 4b). Diverse *cas1* subtypes were detected in Oklahoma grassland microbiomes, i.e., 14 of the 17 subtypes (not IIIA, IIIB, and IIID) were found in both control and warming samples (Fig. 5). The *cas1* subtype composition was not altered much when the soil was heated (Fig. 5).

### Change of *rplB* abundances and *cas1* prevalence in each taxon under soil warming treatment

**Fig. 6:**
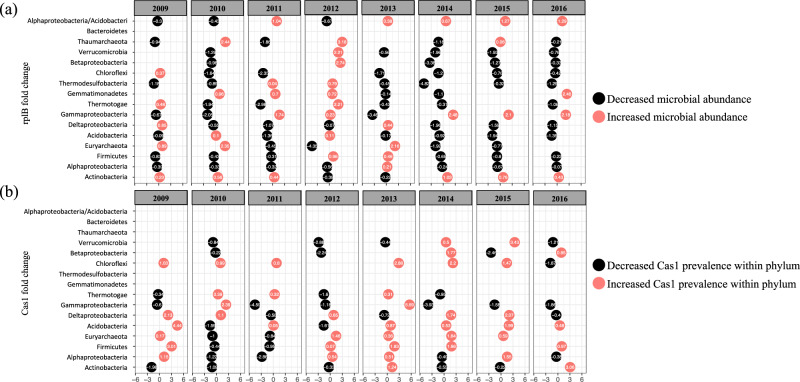
Responses of microbial abundance (*rplB*) and *cas1* prevalence within each major taxon (relative microbial abundance >1%) to 8-year soil warming treatment. Fold change (log2) of *rplB* abundance of each major taxon (relative abundance >1%) (**a**) and the *cas1* prevalence within the corresponding phyla (**b**) in response to the 8-years’ soil warming; All listed major taxa are arranged according to an ascending order of *rplB* abundances. The positive and negative values of log2 transformed fold changes are differentiated by red and black bubbles, respectively.

To assess the general dynamics of microbial communities in response to 8 years of soil warming, we calculated the fold changes of *rplB* abundance (Fig. 6a) and *cas1* prevalence in major soil taxa (Fig. 6b). There was no gradual temporal pattern observed over the 8-year continuous warming. However, the *rplB* abundances of 9 out of 13 major taxa increased in 2012 when the soil warming effect reached a peak though it dropped thereafter (Fig. 6a). The ratio of *rplB* abundances of *Alphaproteobacteria* to *Acidobacteria* was included as an indicator of nutrient availability since *Alphaproteobacteria* are regarded as fast-growing and copiotrophic microbes in contrast to *Acidobacteria*, which are more generally oligotrophic. This ratio gradually increased after 2012 and doubled since 2015 (Fig. 6a). Moreover, *cas1* prevalence tended to increase in warming plots after 2012, even in the taxa with decreased *rplB* abundances, such as *Acidobacteria*, *Chloroflexi*, *Deltaproteobacteria*, *Euryarchaeota* (Fig. 6b), suggesting a preference to CRISPR-Cas carrying microbes.

## Discussion

### Targeted-assembly method gives a high coverage of *cas1* diversity and a reliable host assignment

The conventional approach to studying CRISPR-Cas subtype and host taxonomy is annotating the de-novo assembled contigs [6]. This largely limits the detection ability due to the incomplete assemblies from metagenomes with high sequence complexity such as in soil. To overcome this challenge, we constructed eight Cas1 HMMs to assemble the universal and most conserved Cas protein [1] directly from the metagenomes using Xander. The main advantages of this targeted approach are high coverage of *cas1* sequence diversity, subtype classification, and reliable host assignment.

Targeted assembly using the eight HMMs provided a high coverage of the 17 subtypes of *cas1* as validated with a simulated metagenome. This provides the opportunity to deeply mine the CRISPR-Cas system in more complex microbiomes. *cas1* protein sequences generally clustered according to subtypes which were classified based on the organization of CRISPR-Cas system loci (Fig. 3) [5]. Therefore, the *cas1* protein tree can be used as a template to place the *cas1* protein assemblies and assign the subtypes.

In comparison with the traditional method of annotating *cas1* on the contigs de-novo assembled from the Oklahoma soil metagenomes, the targeted method recovered five times more *cas1* with near full length in addition to the another ~3300 shorter ones, which could be from the less abundant microbes. Analyzing the targeted-assembled *cas1* could provide new opportunities to investigate the diverse CRISPR-Cas system. CRISPR arrays are reported to be horizontally transferred with Cas proteins homologous across different subtypes [49, 50]. No studies, however, specifically investigated the mobility of *cas1* proteins. To validate the *cas1*-based host assignment to the phylum level, we compared the results of mapping the targeted-assembled *cas1* to the de-novo assembled contigs from the Oklahoma soil metagenomes.

Only a small proportion of the targeted-assembled *cas1* nucleotide sequences could be mapped to the de-novo assembled contigs and all were within the *cas1* coding regions, highlighting that the targeted-assembly method can potentially better recover *cas1* diversity with high assembly accuracy. None of the *cas1*-mapped de-novo assembled contigs (*sans* CRISPR-Cas-related genes) were assigned to a different host taxonomy by the targeted and de-novo assembly methods (Supplementary T4). This showed strong evidence that at least in Oklahoma grassland microbiomes, *cas1* protein sequences were less mobile across phyla. This could be due to the high sequence diversity giving distinctive *cas1* features and/or selective usage of CRISPR-Cas system by different phyla. The majority of *cas1*-mapped de-novo assembled contigs were annotated as *Euryarchaeota* and *Thermotogae*, which are also the two most dominant *cas1* hosts (Fig. 4c, Cas1), although they were minor groups in the total microbial community (Fig. 4c, rplB). The targeted-assembly method can retrieve *cas1* even from the less abundant microbes, providing more opportunities to uncover CRISPR-Cas ecology. Comparing with the de-novo assembled contigs is an important step to further validate the assembly accuracy and whether the gene of interest can inform the host taxonomy, especially when studying new habitats.

### Effects of soil warming on *cas1* subtypes and preference in Oklahoma grassland microbiome

*rplB* and *cas1* were targeted-assembled from 48 metagenomes encompassing 8 years of continuous warming of a tallgrass prairie soil in Oklahoma to investigate the dynamics of microbial abundance and *cas1* prevalence in response to soil warming. The microbial abundance of the major phyla increased in 2012 when the heating differential was the highest, and dropped back after 2012 (Fig. 6a). This may reflect a strong resilience of the soil microbiome at the phylum level. In addition, the microbial abundance ratio of *Alphaproteobacteria* to *Acidobacteria* after 2012 gradually rose and doubled after 2015. This ratio has been used to suggest nutrient availability in which fast-growing microbes like *Proteobacteria* are favored over slow-growing bacteria like *Acidobacteria* that are more successful in low nutrient environments [16, 17]. The increasing soil temperature may act as a trigger and slowly induce changes or sequential reactions affecting the other environmental factors, such as pH, moisture, carbon, and nitrogen [51], which may explain the strong negative correlations between soil temperature with pH, and pH with moisture and nitrogen (Supplementary Fig 1). *cas1* host composition, as a result, could be influenced by a combined effect of temperature, pH and moisture, and nutrients (total carbon, total nitrogen, nitrate, and ammonium) (Fig. 4b). Although the overall microbial communities have an ability to recover after environmental perturbation, the warming effect may lead to changes of soil physiochemistry and slowly affect the microbe and *cas1* host composition.

We detected a diverse but similar *cas1* subtype composition in the warming samples and the paired controls. Therefore, there may be a microbial shift within a phylum carrying the same subtypes in response to soil warming. Our previous study at the same sampling site indeed revealed that this warming treatment led to an increasing divergence of microbial composition based on 16S ribosomal RNA genes [15]. A recent study has experimentally demonstrated that CRISPR-Cas systems can be shared between bacterial genera [52]. Replicating and passing the same mature CRISPR-Cas systems among the different and same genera within a phylum may be advantageous for microbial survival under environmental perturbation.

In addition to the need of detecting the CRISPR-Cas dynamics within phyla, CRISPR-Cas system was favored within different microbial taxa, especially in *Euryarchaeota* and *Thermotogae* at our study site. As a result, the total counts of *cas1* mainly reflect the abundance changes of the *cas1*-predominant hosts. Therefore, we calculated the *cas1* prevalence per phylum, which can reveal a preference of CRISPR-Cas system within each host phylum. The prevalence of *cas1* in each taxon tended to increase in response to soil warming. In 2013, 8 out of 10 taxa were increased in *cas1* prevalence, 7 out of 11 taxa in 2014, and 6 out of 9 taxa in 2015 while 4 out of 9 in 2016 (Fig. 6b). The prevalence of *cas1* or CRISPR-Cas system within each taxon also increased in the other major taxa, even in those with decreased microbial abundances after the heating stimuli, such as in *Acidobacteria* and *Firmicutes* (Fig. 6a). These imply that CRISPR-Cas system became more preferred in the major taxa after the largest temperature differential, suggesting more intense interactions with the viruses after soil warming. The higher temperature could increase the virulence of soil phages and induce the release of free phages as more become lytic [53].

Here, we validated and applied the gene targeted-assembly method to detect the CRISPR-Cas subtype diversity and predict *cas1* hosts. Although the soil microbiome is known to have strong resilience to environmental changes, increasing CRISPR-Cas preference was observed in response to 8 years of soil warming. Applying this new method to environmental samples can improve our understanding of the ecological outcomes, and potentially the role of CRISPR-Cas in nature.

## Supplementary information

Supplementary T1

Supplementary File 1

Supplementary T2

Supplementary T3

Supplementary T4

Supplementary Fig 1 Correlation of the environmental attributes.

## Data Availability

The shotgun metagenomic sequences have been deposited in the National Center for Biotechnology Information under the BioProject PRJNA533082. The *cas1* assemblies were submitted to DDBJ/EMBL/GenBank as a Targeted Locus Study project under the accession KDDF00000000, PRJNA551292. The de-novo assembled contigs are available at https://iegst1.rccc.ou.edu/owncloud/index.php/s/mODjEsnzJ8Jxe3s. The eight Cas1 HMMs can be downloaded from Fungene website (http://fungene.cme.msu.edu) and Xander packages are available on GitHub, https://github.com/Ruonan0101/Targeted_Cas1_assembly.
