# Supplementary material for: Targeted assemblies of cas1 suggest CRISPR-Cas’s response to soil warming
Source: ISME J. 2020 Mar 27;14(7):1651–62. doi: 10.1038/s41396-020-0635-1 (PMC7305122; doi:10.1038/s41396-020-0635-1)
Supplement: Supplementary file 1 — Supplementary T1 [file 41396_2020_635_MOESM1_ESM.docx]

**Supplementary information**

**Targeted assemblies of *cas1* suggest CRISPR-Cas’s response to soil warming**

Ruonan Wu^1,2^, Benli Chai^2^, James R. Cole^2,3^, Santosh K. Gunturu^2^, Xue Guo^4,6^, Renmao Tian^4,7^, Ji-Dong Gu^1^, Jizhong Zhou^4,5,6^, James M. Tiedje^2,3*^

1. Laboratory of Environmental Microbiology and Toxicology, School of Biological Sciences, Faculty of Science, The University of Hong Kong, Hong Kong SAR, China

2. Center for Microbial Ecology, Michigan State University, East Lansing, MI, USA

3. Department of Plant, Soil and Microbial Sciences, Michigan State University, East Lansing, MI, USA

4. Institute for Environmental Genomics, Department of Microbiology & Plant Biology, and School of Civil Engineering and Environmental Sciences, University of Oklahoma, Norman, OK, USA

5. Earth and Environmental Sciences, Lawrence Berkeley National Laboratory, Berkeley, California, USA

6. State Key Joint Laboratory of Environment Simulation and Pollution Control, School of Environment, Tsinghua University, Beijing, China

7. Institute for Food Safety and Health, Illinois Institute of Technology, Chicago, IL, USA

* Corresponding author

Tel: (1) 517-353-9021; Fax: (1) 517-353-2917; E-mail: tiedjej@msu.edu

**Supplementary Table 1** Summary of reference genomes in training set.

| No. of. Cas1 | No. of Genomes | Species/Strain Name | Genome Accession No. | Cas1 Subtype | Cas1 Protein Accession No. | Lineage |
| --- | --- | --- | --- | --- | --- | --- |
| 1 | 1 | Candidatus Micrarchaeum acidiphilum ARMAN-1 | MOEG01000020.1 | ArchealII | OJI07266.1 | Candidatus Micrarchaeota |
| 2 | 2 | Deltaproteobacteria | MGPG01000094.1 | CasX | [OGP07440.1](https://www.ncbi.nlm.nih.gov/protein/1084659053) | Proteobacteria |
| 3 | 3 | Parcubacteria group bacterium | KY040242.1 | CasY | APG80665.1 | unclassified Parcubacteria group |
| 4 | 4 | katanobacteria bacterium CG09_39_24 | MOEH01000029.1 | CasY | [OJI08768.1](https://www.ncbi.nlm.nih.gov/protein/1111370281) | unclassified bacteria |
| 5 | 5 | vogelbacteria bacterium CG10_37_50 | MOEJ01000028.1 | CasY | [OJI07454.1](https://www.ncbi.nlm.nih.gov/protein/1111368905) | unclassified bacteria |
| 6 | 6 | Thermosphaera_aggregans_DSM_11486 | NC_014160.1 | IA | [WP_013129675.1](https://www.ncbi.nlm.nih.gov/protein/502894699) | Crenarchaeota |
| 7 | 7 | Vulcanisaeta distributa DSM 14429 | [NC_014537.1](https://www.ncbi.nlm.nih.gov/nuccore/NC_014537.1) | IA | [WP_013336798.1](https://www.ncbi.nlm.nih.gov/protein/WP_013336798.1) | Crenarchaeota |
| 8 | 8 | Vulcanisaeta moutnovskia 768-28 | [NC_015151.1](https://www.ncbi.nlm.nih.gov/nuccore/NC_015151.1) | IA | [WP_013604767.1](https://www.ncbi.nlm.nih.gov/protein/WP_013604767.1) | Crenarchaeota |
| 9 | 9 | Pyrolobus fumarii 1A | [NC_015931.1](https://www.ncbi.nlm.nih.gov/nuccore/NC_015931.1) | IA | [WP_014026048.1](https://www.ncbi.nlm.nih.gov/protein/WP_014026048.1) | Crenarchaeota |
| 10 | 10 | Desulfurococcus amylolyticus DSM 16532 | [NC_018001.1](https://www.ncbi.nlm.nih.gov/nuccore/NC_018001.1) | IA | [WP_014767791.1](https://www.ncbi.nlm.nih.gov/protein/WP_014767791.1) | Crenarchaeota |
| 11 | 11 | Carboxydothermus hydrogenoformans Z-2901 | [NC_007503.1](https://www.ncbi.nlm.nih.gov/nuccore/NC_007503.1) | IB | [WP_011345039.1](https://www.ncbi.nlm.nih.gov/protein/WP_011345039.1) | Firmicutes |
| 12 | 12 | Clostridium novyi NT | [NC_008593.1](https://www.ncbi.nlm.nih.gov/nuccore/NC_008593.1) | IB | [WP_011722692.1](https://www.ncbi.nlm.nih.gov/protein/WP_011722692.1) | Firmicutes |
| 13 | 13 | Desulfotomaculum reducens MI-1 | [NC_009253.1](https://www.ncbi.nlm.nih.gov/nuccore/NC_009253.1) | IB | [WP_011876922.1](https://www.ncbi.nlm.nih.gov/protein/WP_011876922.1) | Firmicutes |
| 14 | 14 | Caldicellulosiruptor saccharolyticus DSM 8903 | [NC_009437.1](https://www.ncbi.nlm.nih.gov/nuccore/NC_009437.1) | IB | [WP_011917894.1](https://www.ncbi.nlm.nih.gov/protein/WP_011917894.1) | Firmicutes |
| 15 | 15 | Thermosipho melanesiensis BI429 | [NC_009616.1](https://www.ncbi.nlm.nih.gov/nuccore/NC_009616.1) | IB | [WP_012056579.1](https://www.ncbi.nlm.nih.gov/protein/WP_012056579.1) | Thermotogae |
| 16 | 16 | Alkaliphilus metalliredigens QYMF | [NC_009633.1](https://www.ncbi.nlm.nih.gov/nuccore/NC_009633.1) | IB | [WP_012063330.1](https://www.ncbi.nlm.nih.gov/protein/WP_012063330.1) | Firmicutes |
| 17 | 17 | Clostridium kluyveri | NC_009706.1 | IB | [WP_012103092.1](https://www.ncbi.nlm.nih.gov/protein/WP_012103092.1) | Firmicutes |
| 18 | 18 | Fervidobacterium nodosum Rt17-B1 | [NC_009718.1](https://www.ncbi.nlm.nih.gov/nuccore/NC_009718.1) | IB | [WP_011994107.1](https://www.ncbi.nlm.nih.gov/protein/WP_011994107.1) | Thermotogae |
| 19 | 19 | Thermotoga sp. RQ2 | [NC_010483.1](https://www.ncbi.nlm.nih.gov/nuccore/NC_010483.1) | IB | [WP_012310886.1](https://www.ncbi.nlm.nih.gov/protein/WP_012310886.1) | Thermotogae |
| 20 | 20 | Chlorobium limicola DSM 245 | [NC_010803.1](https://www.ncbi.nlm.nih.gov/nuccore/NC_010803.1) | IB | [WP_012465419.1](https://www.ncbi.nlm.nih.gov/protein/WP_012465419.1) | Chlorobi |
| 21 | 21 | Chloroherpeton thalassium ATCC 35110 | [NC_011026.1](https://www.ncbi.nlm.nih.gov/nuccore/NC_011026.1) | IB | [WP_012500880.1](https://www.ncbi.nlm.nih.gov/protein/WP_012500880.1) | Chlorobi |
| 22 | 22 | Dictyoglomus thermophilum H-6-12 | [NC_011297.1](https://www.ncbi.nlm.nih.gov/nuccore/NC_011297.1) | IB | [WP_012547158.1](https://www.ncbi.nlm.nih.gov/protein/WP_012547158.1) | Dictyoglomi |
| 23 | 23 | Anoxybacillus flavithermus WK1 | [NC_011567.1](https://www.ncbi.nlm.nih.gov/nuccore/NC_011567.1) | IB | [WP_012574448.1](https://www.ncbi.nlm.nih.gov/protein/WP_012574448.1) | Firmicutes |
| 24 | 24 | Campylobacter fetus subsp. fetus 82-40 | [NC_022759.1](https://www.ncbi.nlm.nih.gov/nuccore/NC_022759.1) | IB | [WP_023384787.1](https://www.ncbi.nlm.nih.gov/protein/WP_023384787.1) | Proteobacteria |
| 25 | 25 | Methylococcus capsulatus str. Bath | [NC_002977.6](https://www.ncbi.nlm.nih.gov/nuccore/NC_002977.6) | IC | [WP_010959993.1](https://www.ncbi.nlm.nih.gov/protein/WP_010959993.1) | Proteobacteria |
| 26 | 26 | Nitrobacter winogradskyi Nb-255 | [NC_007406.1](https://www.ncbi.nlm.nih.gov/nuccore/NC_007406.1) | IC | [WP_011315189.1](https://www.ncbi.nlm.nih.gov/protein/WP_011315189.1) | Proteobacteria |
| 27 | 27 | Pelodictyon luteolum DSM 273 | [NC_007512.1](https://www.ncbi.nlm.nih.gov/nuccore/NC_007512.1) | IC | [WP_011358032.1](https://www.ncbi.nlm.nih.gov/protein/WP_011358032.1) | Chlorobi |
| 28 | 28 | Frankia sp. CcI3 | [NC_007777.1](https://www.ncbi.nlm.nih.gov/nuccore/NC_007777.1) | IC | [WP_011437729.1](https://www.ncbi.nlm.nih.gov/protein/WP_011437729.1) | Actinobacteria |
| 29 | 29 | Rhodoferax ferrireducens T118 | [NC_007908.1](https://www.ncbi.nlm.nih.gov/nuccore/NC_007908.1) | IC | [WP_011466169.1](https://www.ncbi.nlm.nih.gov/protein/WP_011466169.1) | Proteobacteria |
| 30 | 30 | Methylobacillus flagellatus KT | NC_007947.1 | IC | [WP_011478973.1](https://www.ncbi.nlm.nih.gov/protein/499798239) | Proteobacteria |
| 31 | 31 | Deinococcus geothermalis DSM 11300 | [NC_008025.1](https://www.ncbi.nlm.nih.gov/nuccore/NC_008025.1) | IC | [WP_011529383.1](https://www.ncbi.nlm.nih.gov/protein/WP_011529383.1) | Deinococcus-Thermus |
| 32 | 32 | Myxococcus xanthus DK 1622 | [NC_008095.1](https://www.ncbi.nlm.nih.gov/nuccore/NC_008095.1) | IC | [WP_011556934.1](https://www.ncbi.nlm.nih.gov/protein/WP_011556934.1) | Proteobacteria |
| 33 | 33 | Bifidobacterium adolescentis ATCC 15703 | [NC_008618.1](https://www.ncbi.nlm.nih.gov/nuccore/NC_008618.1) | IC | [WP_003811029.1](https://www.ncbi.nlm.nih.gov/protein/WP_003811029.1) | Actinobacteria |
| 34 | 34 | Pseudomonas stutzeri A1501 | [NC_009434.1](https://www.ncbi.nlm.nih.gov/nuccore/NC_009434.1) | IC | [WP_020305857.1](https://www.ncbi.nlm.nih.gov/protein/WP_020305857.1) | Proteobacteria |
| 35 | 35 | Actinobacillus succinogenes 130Z | [NC_009655.1](https://www.ncbi.nlm.nih.gov/nuccore/NC_009655.1) | IC | [WP_012073029.1](https://www.ncbi.nlm.nih.gov/protein/WP_012073029.1) | Proteobacteria |
| 36 | 36 | Leptothrix cholodnii SP-6 | [NC_010524.1](https://www.ncbi.nlm.nih.gov/nuccore/NC_010524.1) | IC | [WP_012346185.1](https://www.ncbi.nlm.nih.gov/protein/WP_012346185.1) | Proteobacteria |
| 37 | 37 | Exiguobacterium sibiricum 255-15 | [NC_010556.1](https://www.ncbi.nlm.nih.gov/nuccore/NC_010556.1) | IC | [WP_012369064.1](https://www.ncbi.nlm.nih.gov/protein/WP_012369064.1) | Firmicutes |
| 38 | 38 | Xanthomonas oryzae pv. oryzae PXO99A | [NC_010717.2](https://www.ncbi.nlm.nih.gov/nuccore/NC_010717.2) | IC | [WP_011407655.1](https://www.ncbi.nlm.nih.gov/protein/WP_011407655.1) | Proteobacteria |
| 39 | 39 | Cellvibrio japonicus Ueda107 | [NC_010995.1](https://www.ncbi.nlm.nih.gov/nuccore/NC_010995.1) | IC | [WP_012488049.1](https://www.ncbi.nlm.nih.gov/protein/WP_012488049.1) | Proteobacteria |
| 40 | 40 | Pelodictyon phaeoclathratiforme BU-1 | [NC_011060.1](https://www.ncbi.nlm.nih.gov/nuccore/NC_011060.1) | IC | [WP_012508386.1](https://www.ncbi.nlm.nih.gov/protein/WP_012508386.1) | Chlorobi |
| 41 | 41 | Streptococcus equi subsp. zooepidemicus | [NC_012470.1](https://www.ncbi.nlm.nih.gov/nuccore/NC_012470.1) | IC | [WP_012678338.1](https://www.ncbi.nlm.nih.gov/protein/WP_012678338.1) | Firmicutes |
| 42 | 42 | Microcystis aeruginosa NIES-843 | [NC_010296.1](https://www.ncbi.nlm.nih.gov/nuccore/NC_010296.1) | ID | [WP_012266061.1](https://www.ncbi.nlm.nih.gov/protein/WP_012266061.1) | Cyanobacteria |
| 43 | 43 | Nostoc punctiforme PCC 73102 | [NC_010628.1](https://www.ncbi.nlm.nih.gov/nuccore/NC_010628.1) | ID | [WP_012409223.1](https://www.ncbi.nlm.nih.gov/protein/WP_012409223.1) | Cyanobacteria |
| 44 | 44 | Escherichia coli K-12 substr. MG1655 | NC_000913.3 | IE | [NP_417235.1](https://www.ncbi.nlm.nih.gov/protein/NP_417235.1) | Proteobacteria |
| 45 | 25 | Methylococcus capsulatus str. Bath | [NC_002977.6](https://www.ncbi.nlm.nih.gov/nuccore/NC_002977.6) | IE | [WP_010960239.1](https://www.ncbi.nlm.nih.gov/protein/WP_010960239.1) | Proteobacteria |
| 46 | 45 | Streptomyces avermitilis MA-4680 = NBRC 14893 | [NC_003155.5](https://www.ncbi.nlm.nih.gov/nuccore/NC_003155.5) | IE | [WP_063773990.1](https://www.ncbi.nlm.nih.gov/protein/WP_063773990.1) | Actinobacteria |
| 47 | 46 | Thermobifida fusca YX | [NC_007333.1](https://www.ncbi.nlm.nih.gov/nuccore/NC_007333.1) | IE | [WP_011292015.1](https://www.ncbi.nlm.nih.gov/protein/WP_011292015.1) | Actinobacteria |
| 48 | 47 | Dehalococcoides sp. CBDB1 | [NC_007356.1](https://www.ncbi.nlm.nih.gov/nuccore/NC_007356.1) | IE | [WP_011309884.1](https://www.ncbi.nlm.nih.gov/protein/WP_011309884.1) | Chloroflexi |
| 49 | 48 | Synechococcus sp. JA-3-3Ab | [NC_007775.1](https://www.ncbi.nlm.nih.gov/nuccore/NC_007775.1) | IE | [WP_011429623.1](https://www.ncbi.nlm.nih.gov/protein/WP_011429623.1) | Cyanobacteria |
| 50 | 28 | Frankia sp. CcI3 | [NC_007777.1](https://www.ncbi.nlm.nih.gov/nuccore/NC_007777.1) | IE | [WP_011434499.1](https://www.ncbi.nlm.nih.gov/protein/WP_011434499.1) | Actinobacteria |
| 51 | 49 | Chromohalobacter salexigens DSM 3043 | [NC_007963.1](https://www.ncbi.nlm.nih.gov/nuccore/NC_007963.1) | IE | [WP_011505542.1](https://www.ncbi.nlm.nih.gov/protein/WP_011505542.1) | Proteobacteria |
| 52 | 50 | Psychromonas ingrahamii 37 | [NC_008709.1](https://www.ncbi.nlm.nih.gov/nuccore/NC_008709.1) | IE | [WP_011769950.1](https://www.ncbi.nlm.nih.gov/protein/WP_011769950.1) | Proteobacteria |
| 53 | 51 | Pseudomonas mendocina ymp | [NC_009439.1](https://www.ncbi.nlm.nih.gov/nuccore/NC_009439.1) | IE | [WP_041772964.1](https://www.ncbi.nlm.nih.gov/protein/WP_041772964.1) | Proteobacteria |
| 54 | 52 | Vibrio cholerae O395 | [NC_009457.1](https://www.ncbi.nlm.nih.gov/nuccore/NC_009457.1) | IE | [WP_001051767.1](https://www.ncbi.nlm.nih.gov/protein/WP_001051767.1) | Proteobacteria |
| 55 | 53 | Geobacter uraniumreducens Rf4 | [NC_009483.1](https://www.ncbi.nlm.nih.gov/nuccore/NC_009483.1) | IE | [WP_011937767.1](https://www.ncbi.nlm.nih.gov/protein/WP_011937767.1) | Proteobacteria |
| 56 | 54 | Marinomonas sp. MWYL1 | [NC_009654.1](https://www.ncbi.nlm.nih.gov/nuccore/NC_009654.1) | IE | [WP_012071209.1](https://www.ncbi.nlm.nih.gov/protein/WP_012071209.1) | Proteobacteria |
| 57 | 55 | Heliobacterium modesticaldum Ice1 | [NC_010337.2](https://www.ncbi.nlm.nih.gov/nuccore/NC_010337.2) | IE | [WP_012281746.1](https://www.ncbi.nlm.nih.gov/protein/WP_012281746.1) | Firmicutes |
| 58 | 56 | Streptomyces griseus subsp. griseus | [NC_010572.1](https://www.ncbi.nlm.nih.gov/nuccore/NC_010572.1) | IE | [WP_012379974.1](https://www.ncbi.nlm.nih.gov/protein/WP_012379974.1) | Actinobacteria |
| 59 | 57 | Geobacter lovleyi SZ | [NC_010814.1](https://www.ncbi.nlm.nih.gov/nuccore/NC_010814.1) | IE | [WP_041243395.1](https://www.ncbi.nlm.nih.gov/protein/WP_041243395.1) | Proteobacteria |
| 60 | 58 | Chlorobium phaeobacteroides BS1 | [NC_010831.1](https://www.ncbi.nlm.nih.gov/nuccore/NC_010831.1) | IE | [WP_012475533.1](https://www.ncbi.nlm.nih.gov/protein/WP_012475533.1) | Chlorobi |
| 61 | 59 | Prosthecochloris aestuarii DSM 271 | [NC_011059.1](https://www.ncbi.nlm.nih.gov/nuccore/NC_011059.1) | IE | [WP_012505967.1](https://www.ncbi.nlm.nih.gov/protein/WP_012505967.1) | Chlorobi |
| 62 | 60 | Anaeromyxobacter sp. K | [NC_011145.1](https://www.ncbi.nlm.nih.gov/nuccore/NC_011145.1) | IE | [WP_012524847.1](https://www.ncbi.nlm.nih.gov/protein/WP_012524847.1) | Proteobacteria |
| 63 | 61 | Rhodospirillum centenum SW | [NC_011420.2](https://www.ncbi.nlm.nih.gov/nuccore/NC_011420.2) | IE | [WP_012568473.1](https://www.ncbi.nlm.nih.gov/protein/WP_012568473.1) | Proteobacteria |
| 64 | 62 | Photobacterium profundum SS9 | NC_006371.1 | IF | WP_011221975.1 | Proteobacteria |
| 65 | 63 | Acidovorax sp. JS42 | [NC_008782.1](https://www.ncbi.nlm.nih.gov/nuccore/NC_008782.1) | IF | [WP_011803949.1](https://www.ncbi.nlm.nih.gov/protein/WP_011803949.1) | Proteobacteria |
| 66 | 64 | Verminephrobacter eiseniae EF01-2 | [NC_008786.1](https://www.ncbi.nlm.nih.gov/nuccore/NC_008786.1) | IF | [WP_011809227.1](https://www.ncbi.nlm.nih.gov/protein/WP_011809227.1) | Proteobacteria |
| 67 | 65 | Actinobacillus pleuropneumoniae serovar 3 | [NC_009053.1](https://www.ncbi.nlm.nih.gov/nuccore/NC_009053.1) | IF | [WP_005600274.1](https://www.ncbi.nlm.nih.gov/protein/WP_005600274.1) | Proteobacteria |
| 68 | 66 | Enterobacter sp. 638 | [NC_009436.1](https://www.ncbi.nlm.nih.gov/nuccore/NC_009436.1) | IF | [WP_012016801.1](https://www.ncbi.nlm.nih.gov/protein/WP_012016801.1) | Proteobacteria |
| 69 | 67 | Yersinia pseudotuberculosis YPIII | [NC_010465.1](https://www.ncbi.nlm.nih.gov/nuccore/NC_010465.1) | IF | [WP_072080408.1](https://www.ncbi.nlm.nih.gov/protein/WP_072080408.1) | Proteobacteria |
| 70 | 68 | Shewanella baltica OS185 | [NC_016901.1](https://www.ncbi.nlm.nih.gov/nuccore/NC_016901.1) | IF | [WP_012197496.1](https://www.ncbi.nlm.nih.gov/protein/WP_012197496.1) | Proteobacteria |
| 71 | 69 | Methylophaga sp. JAM1 | [NC_017857.3](https://www.ncbi.nlm.nih.gov/nuccore/NC_017857.3) | IF | [WP_014707764.1](https://www.ncbi.nlm.nih.gov/protein/WP_014707764.1) | Proteobacteria |
| 72 | 70 | Coprococcus_catus_GD_7 | FP929038.1 | IIA | [CBK78999.1](https://www.ncbi.nlm.nih.gov/protein/291520706) | Firmicutes |
| 73 | 71 | Acidaminococcus intestini RyC-MR95 | [NC_016077.1](https://www.ncbi.nlm.nih.gov/nuccore/NC_016077.1) | IIA | [WP_009016218.1](https://www.ncbi.nlm.nih.gov/protein/WP_009016218.1) | Firmicutes |
| 74 | 72 | Filifactor alocis ATCC 35896 | [NC_016630.1](https://www.ncbi.nlm.nih.gov/nuccore/NC_016630.1) | IIA | [WP_014262093.1](https://www.ncbi.nlm.nih.gov/protein/WP_014262093.1) | Firmicutes |
| 75 | 73 | Bifidobacterium merycicum LMG11341 | NZ_JGZC01000004.1 | IIA | WP_033522168.1 | Actinobacteria |
| 76 | 74 | Bifidobacterium bifidumLMG13200 | NZ_JSEB00000000.1/NZ_JSEB01000005.1 | IIA | WP_047297910.1 | Actinobacteria |
| 77 | 75 | Wolinella succinogenes DSM 1740 | [NC_005090.1](https://www.ncbi.nlm.nih.gov/nuccore/NC_005090.1) | IIB | [WP_011139432.1](https://www.ncbi.nlm.nih.gov/protein/WP_011139288.1) | Proteobacteria |
| 78 | 76 | Legionella pneumophila Paris | [NC_006368.1](https://www.ncbi.nlm.nih.gov/nuccore/NC_006368.1) | IIB | [WP_080019870.1](https://www.ncbi.nlm.nih.gov/protein/WP_080019870.1) | Proteobacteria |
| 79 | 77 | Bifidobacterium bombi DSM 19703 | NZ_ATLK01000001.1 | IIC | WP_044086767.1 | Actinobacteria |
| 80 | 78 | Bifidobacterium callitrichos DSM 23973 | NZ_JGYS01000008.1 | IIC | WP_043165618.1 | Actinobacteria |
| 81 | 79 | Bifidobacterium tsurumiense JCM13495T | NZ_JGZU01000003.1 | IIC | WP_026642919.1 | Actinobacteria |
| 82 | 80 | staphylococcus epidermidis RP62A | [NC_002976.3](https://www.ncbi.nlm.nih.gov/nuccore/NC_002976.3) | IIIA | [WP_002486040.1](https://www.ncbi.nlm.nih.gov/protein/WP_002486040.1) | Firmicutes |
| 83 | 81 | Staphylococcus lugdunensis HKU09-01 | [NC_013893.1](https://www.ncbi.nlm.nih.gov/nuccore/NC_013893.1) | IIIA | [WP_012990653.1](https://www.ncbi.nlm.nih.gov/protein/WP_012990653.1) | Firmicutes |
| 84 | 82 | Synechococcus sp. JA-2-3B'a(2-13) | [NC_007776.1](https://www.ncbi.nlm.nih.gov/nuccore/NC_007776.1) | IIIB | [WP_011432472.1](https://www.ncbi.nlm.nih.gov/protein/WP_011432472.1) | Cyanobacteria |
| 85 | 83 | Stanieria cyanosphaera | NC_019748.1 | IIIB | WP_015191739.1 | Cyanobacteria |
| 86 | 84 | Methanothermobacter thermautotrophicus | NC_000916.1 | IIIC | [WP_010876708.1](https://www.ncbi.nlm.nih.gov/protein/WP_010876708.1) | Euryarchaeota |
| 87 | 85 | Methanosarcina barkeri str. Fusaro | NC_007355.1 | IIIC | WP_048102388.1 | Euryarchaeota |
| 88 | 86 | Methanomethylovorans hollandica DSM 15978 | [NC_019977.1](https://www.ncbi.nlm.nih.gov/nuccore/NC_019977.1) | IIIC | [WP_015324539.1](https://www.ncbi.nlm.nih.gov/protein/WP_015324539.1) | Euryarchaeota |
| 89 | 87 | Rhodobacter capsulatus SB 1003 | [NC_014034.1](https://www.ncbi.nlm.nih.gov/nuccore/NC_014034.1) | IIID | [WP_031321060.1](https://www.ncbi.nlm.nih.gov/protein/WP_031321060.1) | Proteobacteria |
| 90 | 88 | Desulfarculus baarsii DSM 2075 | [NC_014365.1](https://www.ncbi.nlm.nih.gov/nuccore/NC_014365.1) | IIID | [WP_013258512.1](https://www.ncbi.nlm.nih.gov/protein/WP_013258512.1) | Proteobacteria |
| 91 | 89 | Bifidobacterium animalis subsp. lactis DSM 10140 | NC_012815.1 | IU | WP_044059187.1 | Actinobacteria |
| 92 | 90 | Bifidobacterium pullorum LMG 21816 | NZ_JGZJ01000003.1 | IU | WP_043168999.1 | Actinobacteria |
| 93 | 91 | Bifidobacterium tsurumiense JCM13495T | NZ_JGZU01000002.1 | IU | WP_026641772.1 | Actinobacteria |
| outgroup | outgroup | Streptomyces coelicolor A3(2) | NC_003888_3 | transposase | NP_626990.1 | Actinobacteria |

**Supplementary T2** Mapping the targeted assembled cas1 to the respective reference genome by Bowtie.

**Supplementary T3** Host assignments by a conventional method of annotating cas1 on the de-novo assemblies by HMM searching.

**Supplementary T4** Validating cas1-based host assignment method by mapping the targeted assemblies to the de-novo assemblies.

**Supplementary File 1** The method and results of clustering de-novo assembled contigs using average amino acid identity (AAI).

**Supplementary Fig 1** Correlation of the environmental attributes.

The positive correlation coefficients are illustrated as blue bubbles and the negative values are shown as red and yellow bubbles. The significant correlations (p < 0.05) were highlighted by a green star.
