# Supplementary material for: Targeted assemblies of cas1 suggest CRISPR-Cas’s response to soil warming
Source: ISME J. 2020 Mar 27;14(7):1651–62. doi: 10.1038/s41396-020-0635-1 (PMC7305122; doi:10.1038/s41396-020-0635-1)
Supplement: Supplementary file 2 — Supplementary File 1 [file 41396_2020_635_MOESM2_ESM.docx]

**Supplementary File 1**

**Targeted assemblies of *cas1* suggest CRISPR-Cas’s response to soil warming**

Ruonan Wu^1,2^, Benli Chai^2^, James R. Cole^2,3^, Santosh K. Gunturu^2^, Xue Guo^4,6^, Renmao Tian^4,7^, Ji-Dong Gu^1^, Jizhong Zhou^4,5,6^, James M. Tiedje^2,3*^

1. Laboratory of Environmental Microbiology and Toxicology, School of Biological Sciences, Faculty of Science, The University of Hong Kong, Hong Kong SAR, China

2. Center for Microbial Ecology, Michigan State University, East Lansing, MI, USA

3. Department of Plant, Soil and Microbial Sciences, Michigan State University, East Lansing, MI, USA

4. Institute for Environmental Genomics, Department of Microbiology & Plant Biology, and School of Civil Engineering and Environmental Sciences, University of Oklahoma, Norman, OK, USA

5. Earth and Environmental Sciences, Lawrence Berkeley National Laboratory, Berkeley, California, USA

6. State Key Joint Laboratory of Environment Simulation and Pollution Control, School of Environment, Tsinghua University, Beijing, China

7. Institute for Food Safety and Health, Illinois Institute of Technology, Chicago, IL, USA

* Corresponding author

Tel: (1) 517-353-9021; Fax: (1) 517-353-2917; E-mail: [tiedjej@msu.edu](mailto:tiedjej@msu.edu)

**Method**

To generate a unique set of *cas1*-mapped de-novo assembled contigs, we used the single linkage clustering to cluster the de-novo assembled contigs by a cutoff of 100% average amino acid identity (AAI). AAI distance matrix of all the orthologous ORFs between each pair of de-novo assembled contigs was calculated using envenomics package (1).

**Results**

To assess the sequence redundancy of the mapped de-novo assembled contigs and considering the degeneracy of amino acid sequences, the *cas1*-mapped de-novo assemblies were grouped into 25 clusters at 100% AAI. Among the 147 *cas1*-mapped de-novo assemblies, the 110 assigned with the consistent host phyla were detected in 15 clusters. Of the 110 de-novo assembled contigs, there were 102 grouped into seven clusters annotated as *Euryarchaeota* or *Thermotogae* (Supplementary T4), suggesting that these were the two dominant phyla with *cas1* and shared high contig similarity within each phylum.

**Reference**

1. Rodriguez-R LM, Konstantinidis KT. The enveomics collection: a toolbox for specialized analyses of microbial genomes and metagenomes. PeerJ Preprints; 2016. Report No.: 2167-9843.
