# Supplementary figures and images for: Targeted assemblies of cas1 suggest CRISPR-Cas’s response to soil warming
Source: ISME J. 2020 Mar 27;14(7):1651–62. doi: 10.1038/s41396-020-0635-1 (PMC7305122; doi:10.1038/s41396-020-0635-1)

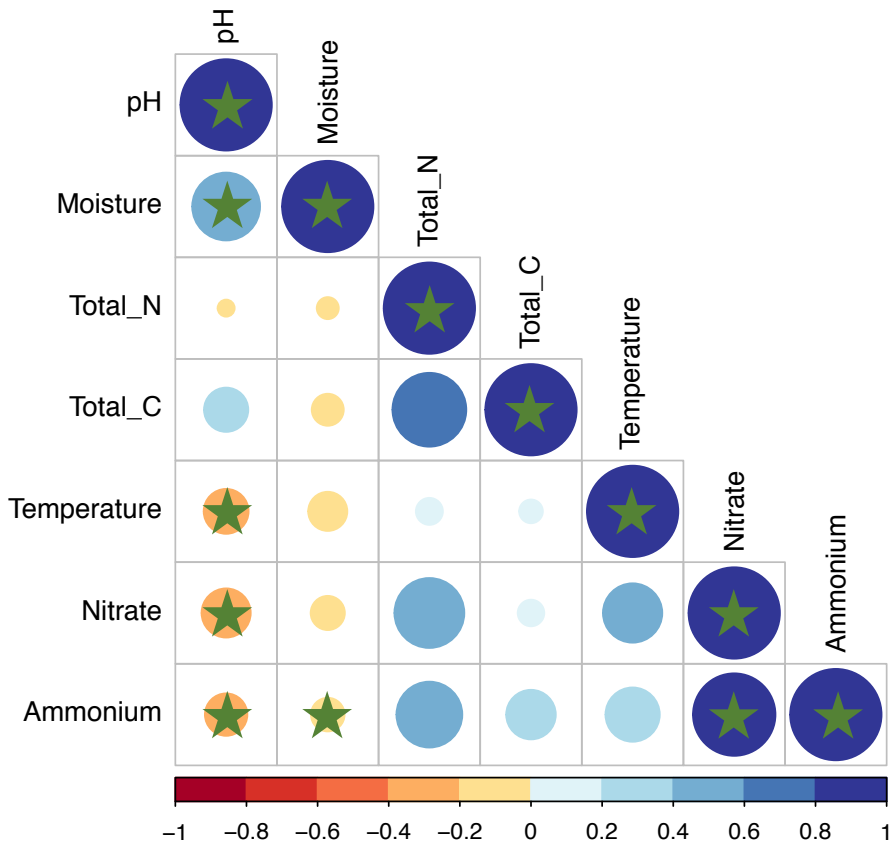

Supplement: Supplementary file 6 — Supplementary Fig 1 Correlation of the environmental attributes. [file 41396_2020_635_MOESM6_ESM.pdf]
